# Association of *CAT* C-262T and *SOD1* A251G single nucleotide polymorphisms susceptible to gastric cancer

**Published:** 2014-12

**Authors:** Shiva Ebrahimpour, Iraj Saadat

**Affiliations:** Department of Biology, College of Sciences, Shiraz University, Shiraz, Iran

**Keywords:** *CAT*, Gastric Cancer, Genetic Polymorphism, Oxidative Stress, * SOD1*

## Abstract

Oxidative stress is known to be one of the major factors involved in the development and progression of cancer. Oxidative stress can occur due to an imbalance between concentrations of reactive oxygen species and antioxidant capacities. Catalase (CAT; OMIM 115500) and superoxide dismutase 1 (SOD1; OMIM 147450) play important roles in the primary defense against oxidative stress. In the present study, we investigated possible associations between polymorphisms of *CAT *C-262T (rs1001179) and *SOD1* A251G (rs2070424) with susceptibility to gastric cancer. This case-control study included 160 gastric cancer patients and 241 age and gender frequency-matched healthy controls. Genotyping was done using PCR-RFLP based method. There were no significant differences in T allele frequencies in patients as compared to the controls in the *CAT* C-262T polymorphism (OR=0.80, 95% CI: 0.52- 1.23, P=0.304). Subjects with AG (OR=0.47, 95% CI: 0.24-0.91, P=0.026) or AG+GG (OR=0.45, 95% CI: 0.23-0.88, P=0.021) genotypes of the rs2070424 polymorphism were at lower risks of developing gastric cancer in comparison with the AA genotype. Our findings showed that there was no significant association between *CAT* C-262T polymorphism and gastric cancer susceptibility. However, we found that the G allele of the *SOD1* A251G polymorphism has protective effects against the risk of gastric cancer.

## INTRODUCTION

Gastric cancer, also known as stomach cancer, can arise in any part of the stomach and may progress to the entire stomach and other surrounding organs such as the esophagus and lymph nodes [1]. Gastric cancer, which is the fourth most common cancer type and the second leading reason of cancer deaths worldwide, is a huge international public health problem [2, 3]. Despite an overall decline in gastric cancer rates in the recent years, this disease still claims 700,000 lives per year [4]. In Iran, it constitutes 22% of all cancer mortalities with an annual report of 5,000 cases [5]. In Fars province (southern Iran), it was found to be the second common cancer in males and the sixth in females [6]. Gastric carcinogenesis is a multi-step and multi-factorial process involving several genetic, epigenetic and environmental risk factors, such as *Helicobacter pylori* (*H. pylori*) infection and reactive oxygen species (ROS) stress [7-9]. The pathogenesis of gastric cancer development is not entirely clear. Superoxide dismutase (SOD) and catalase (CAT) must be involved in neutralizing reactive oxygen species. 

SODs are major antioxidant enzymes which convert superoxides into H_2_O_2 _and O_2_ and are critical in effectively limiting the oxidative burden. Catalase is an endogenous antioxidant enzyme that plays an important role in the primary defense against oxidative stress by converting H_2_O_2_ to O_2_ and H_2_O [10, 11]. Three isoforms of SOD (*SOD1*, CuZn-SOD; *SOD2*, Mn-SOD; and *SOD3*, EC-SOD) have been identified in mammals, among which SOD1 is localized in the intracellular cytoplasmic compartment [11-13]. Oxidative stress is accepted as one of the main factors involved in the development and progression of cancer. Several studies have shown that SOD activity and expression changed significantly in gastric cancer patients [14-16]. 

*CAT* C-262T polymorphism is located 262 bp upstream of the *CAT* transcription site and the association between this polymorphism with the gene expression level shows that the T allele is associated with a higher catalase expression level [17]. Many studies have shown the relationship between genetic polymorphism of *CAT* and *SOD*s genes and multifactorial diseases such as cancers and age-related macular degeneration [18-22]. Due to the above reasons, genetic variations in the *CAT* and *SOD1* genes are suitable candidates that help estimate the genetic susceptibility to gastric cancer. In this study, we investigated the association between gastric cancer and the polymorphisms of *CAT* C-262T and *SOD1* A251G.

## MATERIALS AND METHODS

**Participants:** This case–control study consisted of 160 gastric cancer patients (57 females, 103 males) with a mean age of 57.3 (SD=12.8), all of whom were drafted from the department of chemotherapy, Namazi Hospital, Shiraz (Iran). Two hundred forty-one (75 females, 166 males) healthy individuals, frequency-matched by gender and age with a mean age of 56.8 (SD=10.0), were randomly selected from healthy blood donors. Since the Iranian population is one of the most heterogeneous [23-25], our patients and controls were selected from Persian Muslims (Caucasians) living in Fars province (southern Iran). All individuals were asked to fill in a consent form before the study and complete a self-administered questionnaire regarding their demographic information, family history of cancer in first-degree relatives and tobacco smoking status. Anyone who had at least one family record of cancer in their first-degree relatives was considered as a positive family history case. This study was approved by the Shiraz University ethics committee. Informed consent was obtained from each subject before the study.

**DNA extraction and genotyping analysis: **Whole blood samples were collected in EDTA tube as anticoagulant from patients and the control group members. Immediately after collection, the blood was stored at -20ºC until use. Genomic DNA for PCR was isolated from whole blood using the thawed blood samples by standard protocol [26]. *CAT* C-262T and *SOD*1 A251G genotypes were determined by the PCR-RFLP method as previously described [27, 28]. 

**Statistical analysis: **The Hardy-Weinberg equilibrium was evaluated by performing a Chi-square test. Associations between the polymorphisms’ genotype and the development of gastric cancer were assessed by using odds ratios (OR) and 95% confidence intervals (CIs). Statistical analysis was performed using SPSS statistical software package (SPSS 16.0 for Windows). A probability of p<0.05 was considered as statistically significant.

## RESULTS AND DISCUSSION

General demographic characteristics of gastric cancer patients and the control group are showed in table 1. Data analysis showed significant relationships between smoking status (OR=2.61, 95% CI: 1.65-4.14, P< 0.001) and family history (OR=2.43, 95% CI: 1.41-4.20, P=0.001) with risk of gastric cancer. There were no statistically significant differences between gender (P=0.294) and age (P=0.702) of the cases and the control group (Table 1). Since no statistical difference was observed between genders, the sex groups were pooled.

**Table1:** Selected characteristics of participants of gastric cancer study

**Characteristics**	**Controls (n=241)**	**Cases (n=160)**	**OR**	**95% CI**	**P-value**
**Age (yr) ** **Mean ± SD**	56.8±10.0	57.3±12.8	-	-	0.702
**Gender**					
**Female, N (%)**	75 (31.0)	57 (36.0)			
**Male, N (%)**	166 (69.0)	103 (64.0)	-	-	0.294
					
**Tobacco smoking**					
**Non-smokers**	172 (71.4)	92 (57.5)	1.0	-	-
**Smokers**	45 (18.7)	63 (39.4)	2.61	1.65-4.14	0.000
**Missing**	24 (9.9)	5 (3.1)	-	-	-
					
**Family history**					
**Negative**	153 (63.5)	113 (70.6)	1.0	-	-
**Positive**	25 (10.4)	45 (28.1)	2.43	1.41-4.20	0.001
**Missing**	63 (26.1)	2 (1.3)	-	-	-

The genotypes and allele frequencies of *CAT* C-262T and *SOD1* A251G polymorphisms in the gastric cancer and healthy control groups are shown in Table 2. 

**Table 2:** Distributions of *SOD1* A251G and *CAT* C-262T polymorphisms in cases and controls and risk of gastric cancer

**Polymorphisms**	**Cases (%)**	**Controls (%)**	**OR **	**95% CI**	**P-value**
***SOD1*** ** A251G**					
**AA**	147 (91.8)	202 (83.9)	1.0	-	-
**AG**	13 (8.1)	38 (15.6)	0.47	0.24-0.91	0.026
**GG**	0 (0)	1 (0.41)	-	-	-
**AG+GG**	13	39	0.45	0.23-0.88	0.021
					
**Alleles**					
**A **	307 (95.9)	442 (91.7)	1.0	-	-
**G **	13 (4.1)	40 (8.3)	0.46	0.24-0.89	0.021
***CAT*** ** C-262T**					
**CC**	105 (65.6)	147 (61)	1.0	-	-
**CT**	49 (30.6)	86 (35.7)	0.80	0.52-1.23	0.304
**TT**	6 (3.7)	8 (3.3)	1.05	0.35-3.11	0.930
**CT+TT**	55	94	0.82	0.54-1.24	0.348
					
**Alleles**					
**C **	259 (80.9)	380 (78.8)	1.0	-	-
**T **	61 (19.1)	102 (21.2)	0.88	0.62-1.25	0.470

The genotypic frequencies for the two polymorphisms among the control group members were in Hardy–Weinberg equilibrium (For *CAT* C-262T polymorphism: χ^2^=1.19, df=1, P>0.05; For *SOD1* A251G polymorphism: χ^2^=0.39, df=1, P>0.05). 

More common alleles of both polymorphisms were considered as reference alleles. Logistic regression analysis showed no significant association between *CAT* C-262T polymorphism (CT vs CC: OR=0.8, 95% CI: 0.52-1.23, P=0.304; TT vs CC: OR=1.05, 95% CI: 0.35-3.11, P=0.93) and susceptibility to gastric cancer (Table 2), while other analyses showed significant differences between cases and controls in the *SOD1* A251G polymorphism (AG vs AA: OR=0.47, 95% CI: 0.24-0.91, P=0.026). Analysis also showed that the G allele of *SOD1* decreased the risk of gastric cancer significantly by as much as 0.46 (OR=0.46, 95% CI: 0.24-0.89, P=0.021) (Table 2). 

We also investigated the coincidence effect of *CAT* C-262T and *SOD1* A251G polymorphisms and susceptibility to gastric cancer (Table 3). Analysis showed a significant relationship between genotypes AG+GG of *SOD1* and genotypes CT+TT of *CAT* and the risk of gastric cancer (OR=0.29, 95% CI: 0.09-0.89, P=0.03). 

**Table 3:** Coincidence effect of *CAT* C-262T and *SOD1* A251G polymorphisms and susceptibility to gastric cancer

***SOD1***	***CAT***	**Cases**	**Control** **s**	**OR**	**95% CI**	**P-value**
**AA**	**CC**	96	126	1.0		-
**AA**	**CT+TT**	51	76	0.88	0.56-1.37	0.574
**AG+GG**	**CT+TT**	4	18	0.29	0.09-0.89	0.030
**AG+GG**	**CC**	9	21	0.56	0.24-1.28	0.172

Epidemiological evidence shows that gastric carcinogenesis results from gene-environmental interactions [29]. SOD and CAT are major antioxidant enzymes which play vital roles in the clearance of ROS in vivo and can either promote or suppress tumor formation in human gastric mucosa [14-16]. In conclusion, the present study showed that *CAT* C-262T is not a risk factor for gastric cancer in itself. Our data provides evidence that the G allele of *SOD1* A251G polymorphism is associated with a decreased risk of gastric cancer in the Iranian population. 

Our study might not have had the sufficient power to detect an association between genetic polymorphisms of *CAT* and *SOD1* genes and gastric cancer, because several other single nucleotide polymorphisms for *CAT* (in addition to C-262T) and *SOD1* (in addition to A251G) in humans were not studied. Since the sample size and risk factors investigated in the present study were limited, and several genetic and environmental factors are usually involved in carcinogenesis, our conclusion remains to be further confirmed by studies of larger sample sizes and more risk factors. Previously it has been reported that ethnicity may influence the observed associations in multifactorial disease [30]. Therefore replication of the present study in other races and regions is highly recommended.

## Notes

**Acknowledgements: **The authors are indebted to the participants for their close cooperation. This study was supported by Shiraz University.
